# Potentiometric Determination of Maprotiline Hydrochloride in Pharmaceutical and Biological Matrices Using a Novel Modified Carbon Paste Electrode

**DOI:** 10.3390/s22239201

**Published:** 2022-11-26

**Authors:** Josip Radić, Dorotea Perović, Ema Gričar, Mitja Kolar

**Affiliations:** 1 Department of Environmental Chemistry, Faculty of Chemistry and Technology, University of Split, R. Boškovića 35, 21000 Split, Croatia; 2 Department of Chemistry and Biochemistry, Faculty of Chemistry and Chemical Technology, University of Ljubljana, Večna pot 113, 1000 Ljubljana, Slovenia

**Keywords:** carbon paste electrode (CPE), modifications, potentiometry, maprotiline, drug analyses

## Abstract

Potentiometry with membrane selective electrodes is preferable for measuring the various constituents of pharmaceuticals. In this work, carbon paste electrodes (CPE) were prepared, modified, and tested for the determination of maprotiline hydrochloride, which acts as an antidepressant. The proposed CPE was based on an ionic association complex of maprotiline-tetraphenylborate, 2-nitrophenyloctyl as a binder, and sodium tetraphenylborate as an ionic lipophilic additive. The optimized composition improved potentiometric properties up to theoretical Nernst response values of −59.5 ± 0.8 mV dec^−1^, in the concentration range of maprotiline from 1.6 × 10^−7^ to 1.0 × 10^−2^ mol L^−1^, and a detection limit of 1.1 × 10^−7^ mol L^−1^. The CPE provides excellent reversibility and reproducibility, exhibits a fast response time, and is applicable over a wide pH range. No significant effect was observed in several interfering species tested. The proposed electrode was used for the precise determination of maprotiline in pure solutions, urine samples, and a real sample—the drug Ludiomil.

## 1. Introduction

Depression is one of the leading health problems in the modern world today [1], affecting not only the quality of life [2] but also the financial viability and stability of society as a whole [3]. People suffering from depression or other mental disorders are less able to work, even with the necessary medication. In addition, depression is closely related to the progression of many other diseases [4,5,6,7]. Although numerous studies have already been published on the impact of the COVID19 pandemic [8,9,10,11], much time will pass before all the consequences on peoples’ mental and physical health can be considered. The pandemic is not yet under optimal control, and the world faces additional problems; one of the probable consequences will be the increased consumption of antidepressants [12,13,14], and thus the possibility of their abuse.

One of the most commonly used antidepressants is maprotiline hydrochloride (MAP, (CAS: 10347-81-6)), which is chemically described as 3-(9,10-dihydro-9,10-ethanoanthracen-9-yl)-methylpropyl amine hydrochloride with a molecular formula of C_20_H_23_N·HCl and a molar mass of 313.86 g mol^−1^. It is a white, poorly water-soluble powder and is used to treat depression, major depression and depressive episodes associated with bipolar disorder, and anxiety associated with depression [15]. It is one of the second-generation tetracyclic antidepressants [16]. On average 150 to 225 mg MAP per day per patient is prescribed. However, only 3–4% is excreted in unchanged form in urine or feces [17]. Therefore, the expected unmetabolized concentration of MAP in human urine is 8.1 × 10^−6^ to 2.2 × 10^−5^ mol L^−1^. The importance of its use in human treatment has led to the development of a variety of analytical methods for its determination, including high-performance liquid chromatography [18], liquid chromatography [19,20], reversed-phase ultra-fast liquid chromatography [21], either high [22] or ultra-high [23] performance liquid chromatography coupled to mass spectrometry with supported liquid extraction, either gas or gas-liquid chromatography coupled to mass spectroscopy, capillary electrophoresis, and spectrophotometry [24].

Compared with these powerful modern techniques, potentiometry as one of the techniques of electrochemical analysis based on the measurement of potential difference under equilibrium conditions (at zero current), is incomparably cheaper and does not require additional pretreatment steps and particularly expensive operational staff [25,26,27,28,29].

Ion-selective electrodes (ISEs), as one of the types of electrochemical sensors in potentiometry, produce a potential difference according to the Nernst equation when immersed in a solution of free ions of a certain type. ISEs exhibit a relatively high degree of selectivity for a particular type of ion in solution. Their major advantage is that they respond rapidly to the slightest change in the concentration of the test solution. ISEs are widely used primarily because of their simplicity, are relatively inexpensive, and significantly reduce analysis time compared to other techniques [30]. For the determination of the active ingredients of various pharmaceuticals, ISEs are usually prepared by incorporating the electroactive substances into polyvinyl chloride (PVC)-based membranes [3,24,31,32,33,34,35,36] or carbon paste membranes [35,37,38,39,40,41,42,43,44,45,46,47]. Compared to PVC-based membranes, CPEs are not only faster and easier to fabricate, but also offer easier surface renewal, almost imperceptible ohmic resistance, and certainly more opportunities to modify the membrane composition to improve measurement selectivity [48,49]. To date, two papers have been published on the development of PVC-based electrodes for the determination of MAP [3,24], but none on the fabrication of CPE. Since PVC-based ion-selective electrodes generally have certain shortcomings compared to CPE [38,49,50], the aim of this work was to describe the development and application of modified carbon paste electrodes (mCPE) for the selective and accurate potentiometric determination of MAP, which has not yet been fabricated according to the authors’ knowledge.

## 2. Materials and Methods

### 2.1. Chemicals and Reagents

Commercially available chemicals of high purity were used in this work. Double distilled water was used during the experiments.

Sodium hydroxide anhydrus, glacial acetic acid, hydrochloric acid, iron(III) nitrate nonahydrate, potassium nitrate, copper(II) nitrate trihydrate, and silver nitrate were purchased from Kemika (Croatia). Calcium nitrate tetrahydrate, ammonium chloride, acetylsalicylic acid, phosphotungstic acid hydrate (PTA), L-Arginine, L-Cysteine, tetrabutylammonium tetraphenylborate (TBATPB), bis(2-ethylhexyl) phthalate (DOP), tris(2-ethylhexyl) phosphate (TEPh), and α-lactose monohydrate were purchased from Sigma-Aldrich (Germany). Trihexyphenidyl hydrochloride, dibutylphthalate (DBP), MAP, and paraffin oil (PO) were obtained from Sigma (USA). Lithium nitrate, sodium tetraphenylborate (NaTPB), magnesium nitrate hexahydrate, cobalt(II) nitrate hexahydrate, aluminum nitrate nonahydrate, zinc nitrate hexahydrate, glucose, galactose, and fructose were purchased from Merck (Germany). Bis(2-ethylhexyl) adipate (DEHA) and phosphomolybdic acid hydrate (PMA) were obtained from VWR International BVBA (Belgium). Timrex KS 44 graphite flakes were purchased from Imerys Graphite & Carbon (Switzerland), ammonium reineckate (AR) from Acros Organics BVBA (China), sodium acetate anhydrous from Gram-mol (Croatia) and 2-nitrophenyl octyl ether (NPOE) from Fluka (Switzerland). Paracetamol was a gift from Galenic laboratory Split-Dalmatia County Pharmacy (Croatia), while Ludiomil (25 mg/tablet) was obtained from a pharmaceutical company Pliva (Croatia).

All solutions were prepared by dissolving the appropriate amount of the chemical in a 1.5 × 10^−2^ M acetate buffer solution with a pH of 4.5, except for the solutions used to prepare the ion-association complex (IAC), in which the chemicals were dissolved in double distilled water.

### 2.2. Apparatus and Measurements

Potentiometric measurements were carried out by using Lab pH meter inoLab^®^ pH 7110 (WTW, Weilheim, Germany). Model InLab Reference—51,343,190 (Mettler-Toledo GmbH, Schwerzenbach, Switzerland) was used as an external Ag/AgCl electrode. As in the previously published material [38], the authors performed all potentiometric measurements in an electrochemical cell as follows:

Ag/AgCl (saturated)//sample solution/carbon paste/stainless steel rod.

Electrode InLab Expert Pro (Mettler Toledo GmbH, Greifensee, Switzerland) was used as pH electrode, while pH/mV meter model Seven Easy (Mettler-Toledo, GmbH, Schwerzenbach, Switzerland) was used for pH measurements and adjustments. EcoStir (DLAB Scientific Co., Ltd., Beijing, China) was used as magnetic stirrer. 

CHN analyses were performed on a Perkin-Elmer CHN Analyzer 2400 II.

### 2.3. Preparation of the Ion-Association Complexes

**Figure 1:**
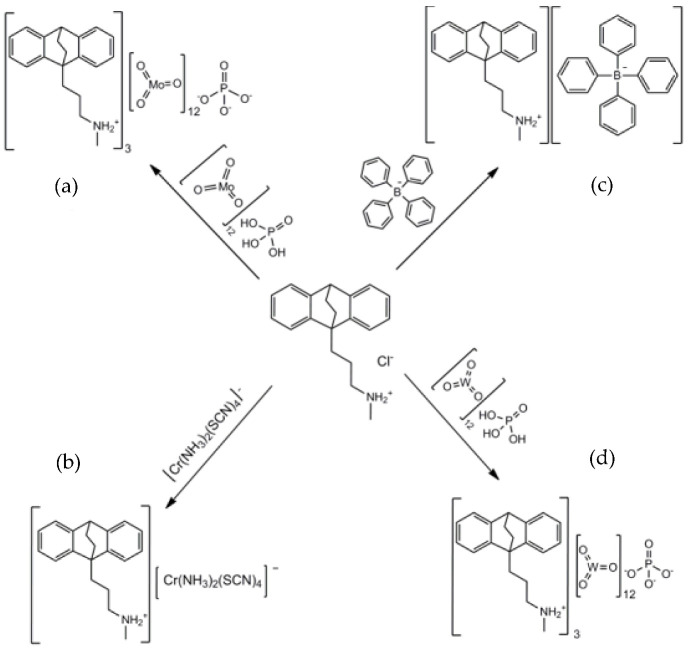
Proposed reaction between the MAP and different ion pairing agents to obtain ion-association complexes: (**a**) MAP_3_-PM; (**b**) MAP-R; (**c**) MAP-TPB; (**d**) MAP_3_-PT.

Stoichiometric volumes of 1.0 × 10^−2^ M MAP solution were mixed with corresponding volumes of 1.0 × 10^−2^ M precipitating reagent solutions (NaTPB, PTA, PMA, AR) to obtain the poorly water-soluble precipitates. The sensing materials of CPE obtained in this way were maprotiline-tetraphenylborate (MAP-TPB), maprotiline-phosphotungstate (MAP_3_-PT), maprotiline-phosphomolybdate (MAP_3_-PM), and maprotiline-reineckate (MAP-R), commonly referred to in the literature as ion-association complexes (Figure 1).

The CHN analysis confirmed the combination rate of reagents to form IAC and the theoretical value of the mass ratio of C, H, and N in each of precipitated materials (Table 1).

### 2.4. Preparation of CPE

A previously prepared and homogenized paste of various proportions of graphite powder, various inert binders, IAC, and ionic additives was gradually pressed into the Teflon body of the electrode with a working diameter of 2 mm, the preparation of which was previously described in detail by Radić et al. [38].

### 2.5. Procedures

Each prepared CPE was preconditioned by soaking in a 1.0 × 10^−7^ M MAP solution for 30 min before use. The potential was measured by CPE and reference Ag/AgCl electrode immersion in a series of MAP working solutions from lower to higher concentrations covering range from 2.5 × 10^−8^ to 1.0 × 10^−2^ mol L^−1^ with continuous stirring. After each immersion and obtaining stable potentiometric readings within change of ±1 mV min^−1^, the potential value was noted. Calibration curves of potential dependence versus negative logarithm of the MAP solution concentrations $\left(-log\left[MAP\right]=pMAP\right)$ were constructed using the linear regression method and the LINEST function of the Microsoft Excel 2016 program.

### 2.6. Determination of Selectivity

Selectivity represents the ability of the sensor to develop a signal on only one target analyte, i.e., it is the sensor’s ability to ensure that similar analytes do not give identical signals.

In this work, the optimized CPE selectivity was examined using the matched potential method (MPM), since it is the only one of all the methods recommended in the literature that does not depend on the Nikolsky-Eisenman equation, as well as neither the pole nor the charge value of the examined and the interfering species. 

To determine electrode selectivity, in the first step, a volume of 0.5 mL of a 1.0 × 10^−3^ M solution of MAP was added ten times in succession to 50 mL of the working solution MAP with a concentration of 1.0 × 10^−5^ mol L^−1^ (initial MAP solution), i.e., until the overall desired concentration of MAP solution of 1.0 × 10^−4^ mol L^−1^ was reached. After each addition, the determined stable potential was recorded.

After that, solutions with a concentration of 0.1 mol L^−1^ (applies to all interfering substances except trihexyphenidyl hydrochloride, whose prepared concentration was 0.01 mol L^−1^) of the tested interfering substances were added in a series of initial MAP solutions until an “overlap” with one of the previously recorded potentials from the first step is observed, i.e., up to one of the same potential values previously recorded in the first step.

Finally, the selectivity coefficient for each tested interfering species was calculated according to the equation:

(1) $$K_{A,B}^{pot}=\frac{a_{MAP}^{'}-a_{MAP}}{a_{INT}}$$

where $a_{MAP}^{'}$ is the overall MAP activity after adding, $a_{MAP}$ is MAP activity in initial solution, and $a_{INT}$ is the activity of an interfering substance after adding.

### 2.7. Determination of Maprotiline in Tablet and Human Urine Samples

This paper presents the determination of maprotiline in real samples using the standard addition method and potentiometric titration. 

Ten Ludiomil tablets (25 mg of MAP per tablet) were ground and transferred to 250 mL of acetate buffer solution, which had previously been heated to 37 °C. After intensive shaking, cooling to room temperature and filtration, the theoretically expected concentration of MAP in the filtrate should be 3.2 × 10^−3^ mol L^−1^, from which further tablet aliquots with lower expected concentrations of MAP (TA) were further prepared (1.0 × 10^−4^, 1.0 × 10^−5^, 1.0 × 10^−6^ mol L^−1^).

Equivalent volumes of 1.0 × 10^−3^ M MAP solution were added to urine samples (taken from an adult male that was not prescribed MAP therapy) to simulate MAP concentrations expected in unmetabolized form in patient urine. Finally, after dilution with acetate buffer, spiked urine sample aliquots with expected MAP concentrations (SUSA) of 1.0 × 10^−4^, 1.0 × 10^−5^, and 1.0 × 10^−6^ mol L^−1^ were obtained.

Small amounts of the standard solutions with a MAP concentration one decade higher than the concentration in TA or SUSA were added successively to the each of prepared TA and SUSA. After each addition, the potential was recorded. The obtained experimental values were recalculated according to a recently published work [38], where the expression $\frac{c_{s}\;\times \;V_{s}}{V_{A}}$ is referred to as the independent variable and the dependent variable as $10^{\frac{E}{S}}\;\times \;\left(V_{A}+V_{s}\right)$ of equation of a straight line. The concentration of the standard MAP solution, the sum of the increments of the standard MAP solution, the initial volume of the tested aliquot, the recorded potential after each increment of the standard MAP solution, and the slope of the calibration curve were denoted by $c_{s},\;V_{s},V_{A},\;E,$ and S, respectively. The intercept of independent variable axis (y=0) of the equation of a straight line represented a negative value of the MAP concentration in the tested aliquots ($-c_{A}$).

Each of the prepared TA and SUSA was titrated against a standard solution of NaTPB and PMA, using as indicator electrode a mCPE no.6, which has given the best response characteristics to MAP of all the electrodes prepared. The zero point of the second derivative ($\frac{d^{2}E}{dV^{2}}$) of the E−V potentiometric titration curve is the inflection point, which represents the equivalent volume, i.e., the drug concentration in the tested aliquot.

## 3. Results and Discussion

### 3.1. Optimization of the CPE Composition

Maprotiline, whose pK_a_ value [51] is 10.5, is a substance that should behave as a monovalent species in aqueous solutions with neutral and acidic pH. It is evident that it has a strong affinity for forming ion pairs with oppositely charged ions, since maprotiline can be protonated at several different active sites in its chemical structure, according to Gonçalves et al. [15]. As such, it readily forms poorly water-soluble IACs with various precipitation reagents.

Subsequently, the influences of the amount and type of IAC, binders, and lipophilic additives on the response of CPE were studied first. 

#### 3.1.1. Selection and Optimization of Liquid Binder

The liquid binder has the role of connecting carbon particles and gives certain chemical-physical and electrochemical properties to the paste itself. The binder should be insoluble in the test solution so that the electrodes do not disintegrate in contact with the test solution; it should have a low vapor pressure that ensures mechanical stability and a long service life of the electrode system, and it should be electrochemically inactive to prevent high background currents and changes in material composition. Along with the dielectric constant of the binder, the hydrophobicity or lipophilicity of the carbon paste (which results from the selected binder) are the most important characteristics that determine the other chemical-physical properties of CPE [48,52]. In this work, the effect of different binders (DBP, DOP, DEHA, PO, TEPh, NPOE) was evaluated (Table 2, mCPE 1-6). The electrode with NPOE as binder showed improved potentiometric properties compared with the other binders examined, especially compared to membranes with DEHA or PO. This implies that NPOE exhibits an optimal ability (of all investigated) to extract MAP ions from the aqueous solution to the organic phase of paste.

#### 3.1.2. Study of IAC—Various Amount and Type

The choice of IAC as the sensing material has one of the main influences on the selectivity of the electrode [47]. Electrodes with no IAC in their composition showed a significantly lower sensitivity to maprotiline; the significant role of IAC in the response characteristic of the CPE is confirmed. For this reason, ion-association complexes were precipitated and incorporated in paste composition as follows: MAP-TPB, MAP_3_-PT, MAP_3_-PM, and MAP-R, whose chemical composition was confirmed by CHN elemental analysis (Table 1). 

The results of the research showed that the optimal value of the mass ratio of IAC in the paste was 2.0%, and that among the four tested IACs, the electrode with the paste containing MAP-TPB as a sensing material showed the best response characteristics (Table 2, mCPE 6-17). In addition, all prepared electrodes containing either paraffin oil as binder or MAP-R as IAC in the membrane composition gave almost undetectable responses.

#### 3.1.3. Role and Selection of Lipophilic Additive

Out of the two ionic additives studied, the presence of 0.1% NaTPB increased the CPE sensitivity from −53.4 ± 1.6 to a Nernst slope of −59.5 ± 0.8 mV dec^−1^ with a wider linear dynamic range, lower detection limit, and better regression coefficient of the calibration curve. Further increasing the amount of NaTPB in the paste saturated the membranes with salt and worsened the response characteristics, as evidenced by the fact that the electrodes then exhibited a super Nernstian slope. The addition of TBATPB to the membrane did not improve the response of the electrodes to maprotiline. Moreover, membranes in which TBATPB was added as an ionic additive did not show improved responses compared to electrodes without an ionic additive.

**Figure 2:**
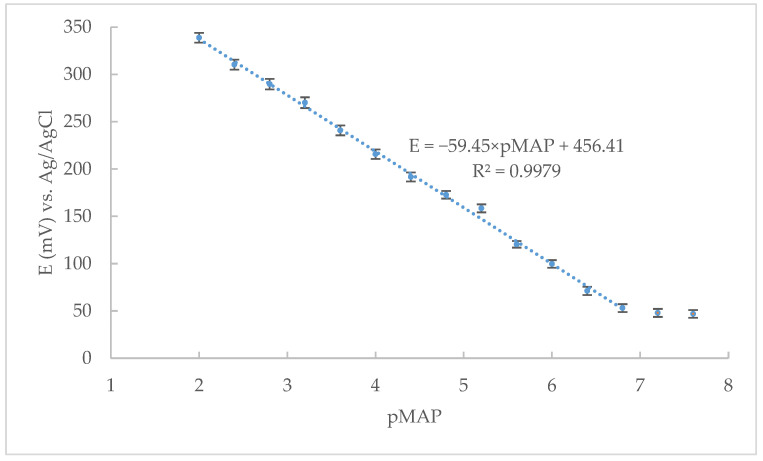
Calibration curve of the optimized CPE for maprotiline potentiometric determination (eleven replicates).

Finally, electrodes containing 48.9% graphite, 49.0% DBP as binder, 2.0% MAP-TPB as IAC, and 0.1% NaTPB as an ionic additive represented the optimal potentiometric response characteristics out of all tested CPE (Figure 2).

### 3.2. Potentiometric Characteristics of Optimized CPE

#### 3.2.1. pH Study

**Figure 3:**
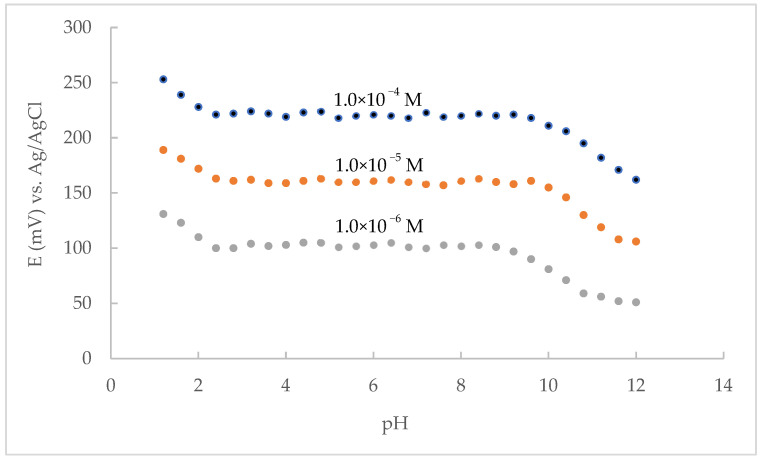
Effect of the pH on the electrode response characteristics.

The effect of pH on the optimized CPE response characteristics was studied by changing the pH of the aqueous solution from 1.2 to 12.0 by adding small amounts of sodium hydroxide or hydrochloric acid solution (1 mol L^−1^) for MAP concentrations of 1.0 × 10^−4^, 1.0 × 10^−5^, 1.0 × 10^−6^ mol L^−1^ (Figure 3). The data showed that the response of studied CPE is constant over a wide pH range from 2.4 to 9.6. Outside the optimized range in acidic media, an increase in the potential value was observed. This can be attributed to the penetration of hydronium ions into the surface of the CPE, i.e., the response of the electrode to hydronium ions instead of that of maprotiline. Considering the pK_a_ value of maprotiline, the lower potential values obtained at pH values above 10 are somehow logical and to be expected, since around this pH range a certain proportion of maprotiline is already in the form of a neutral amine (free base). By further increasing the pH value, the concentration of the maprotiline cation further decreases, which leads to a decrease in potential and a loss of electrode sensitivity to maprotiline. 

#### 3.2.2. Dynamic Response Time, Reversibility, and Reproducibility of CPE

**Figure 4:**
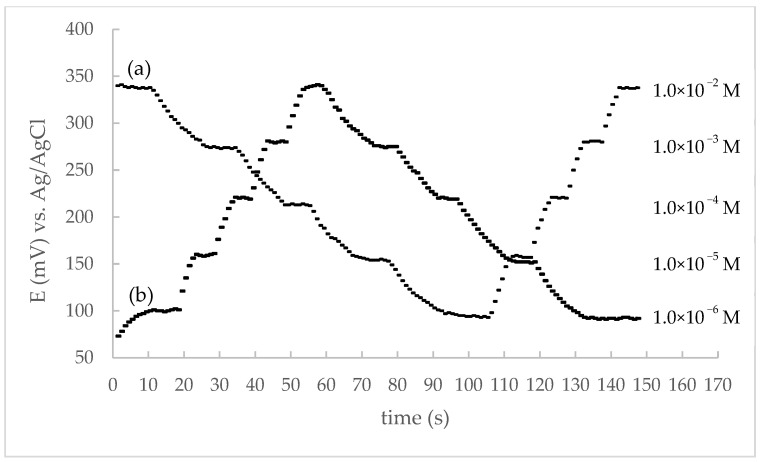
Dynamic response time and reversibility of proposed CPE (**a**)-descending and then ascending order, (**b**) ascending and then descending order of MAP concentration).

The dynamic response time is the time required for the sensor to achieve a stable potential change of ±1 mV from its final value [53]. It was determined by varying the maprotiline concentration from 1.0 × 10^−6^ to 1.0 × 10^−2^ mol L^−1^ and recording the change in potential values over time (Figure 4). At maprotiline concentrations from 1.0 × 10^−5^ to 1.0 × 10^−2^ mol L^−1^, the electrode reached a stable potential in less than 5 s, whereas the response time at a concentration of 1.0 × 10^−6^ mol L^−1^ was around 10 s. 

Reversibility was determined by immersing the optimized CPE in a series of solutions, each with 10-fold higher (ascending order) or lower (descending order) maprotiline concentration compared to the previous studied solution and measuring the potentials. The reversibility of the electrode response can be clearly seen in Figure 4. However, the memory effect phenomenon was also observed, since in descending order the average time required to establish a stable potential was about 15 to 20 s. This can be explained by the fact that maprotiline ions of higher concentrations remain on the surface of the membrane and thus occupy free sites, as the electrode needs more time to detect the potential change of the solution at lower concentrations.

Reproducibility was investigated by preparing nine identically composed optimized electrodes and constructing a calibration curve. The average values and deviations of the slope, detection limit, and linear dynamic range showed high reproducibility as they were −59.5 ± 0.8 mV dec^−1^, 1.1 × 10^−7^ mol L^−1^ and 1.6 × 10^−7^–1.0 × 10^−2^ mol L^−1^, respectively.

#### 3.2.3. Interference Study

The selectivity coefficient is one of the most significant ISE parameters, whereby lower value of the selectivity coefficient implies that the electrode can determine the target species without significant influence of interferents. 

From potential interfering chemical species, those were examined that are most often used as pharmaceutical excipients or those that are the most abundant in urine [37,44,54]. Additionally, the interfering influence of the essential amino acid arginine, non-essential amino acid cysteine, non-steroidal anti-inflammatory drugs, as well as the antidepressant trihexyphenydil hydrochloride, were examined. The estimated coefficient selectivity values are shown in Table 3.

Since most of the selectivity coefficient negative logarithm values $\left(-logK_{MAP,\;INT}^{pot}\right)$ are higher than 3.0, it can be assumed that the optimized CPE is at least 1000 times more selective towards maprotiline than any interfering ion studied. From all this, it is evident that the proposed CPE can be used for the unobstructed determination of maprotiline concentration in presence of foreign ions.

Additionally, in this study, no interferences from nitrates or chlorides, which are acidic residues of the salts studied, were observed. On the other hand, if the presence of ionic species of acetate buffer in the tested pure solutions was considered a potential interfering species, the results of this study confirm that they do not have a significant influence on the selective determination of MAP using mCPE.

### 3.3. Analytical Applications

**Figure 5:**
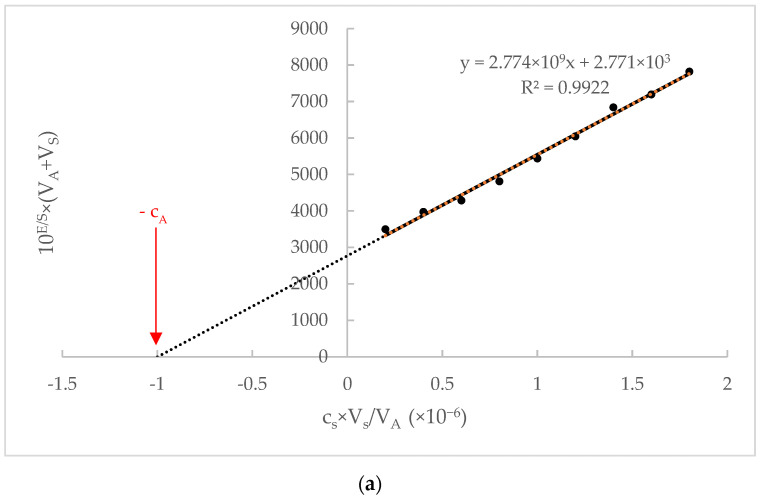

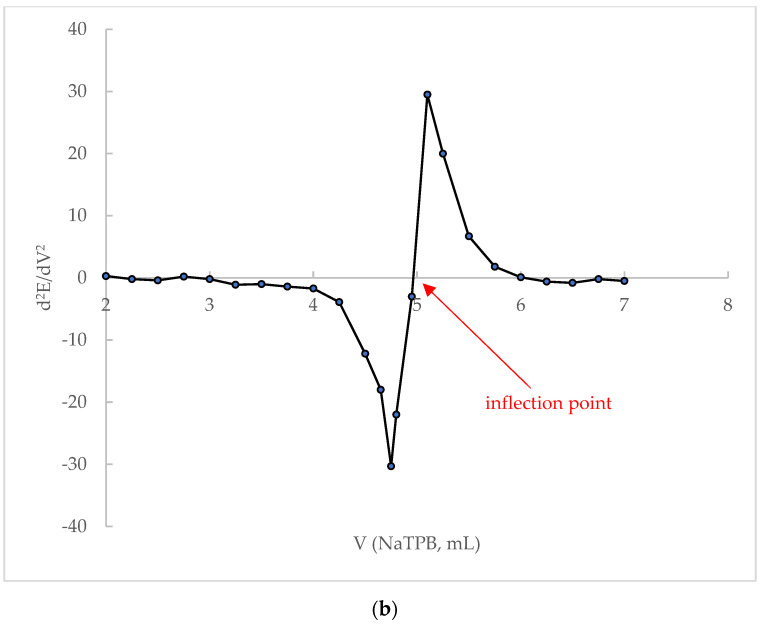
(**a**). Representative graph of the standard addition method application; 1.0 × 10^−5^ mol L^−1^ solution of MAP as spike (standard adding solution) was added to a TA of expected MAP concentration of 1.0 × 10^−6^ mol L^−1^. (**b**). Example application of the second order derivate potentiometric titration curve; 50.0 mL of SUSA of expected MAP concentration of 1.0 × 10^−5^ mol L^−1^ titrated with 1.0 × 10^−4^ mol L^−1^ NATPB solution.

The proposed CPE was examined to determine MAP by using the standard addition method and potentiometric titration by using second derivation in pure solutions, Ludiomil tablets, and aliquot of spiked urine samples. Examples of the processing results obtained using the standard addition method and potentiometric titration are shown in Figure 5a,b. 

From the results presented in Table 4, it is evident that this electrode can be a very useful tool for MAP determination, since obtained results assay has shown agreement between expected and experimental data. It is necessary to note that the proposed electrode gives precise results with low standard deviations, even at lower concentrations of MAP that can realistically be expected in the aliquots of the patient urine samples. 

Greater precision is noticeable using the proposed standard addition method compared to potentiometric titration, especially when determining MAP concentration lower than 1.0 × 10^−5^ mol L^−1^.

Table 5 shows the comparison of the potentiometric properties of the electrode proposed in this work with the characteristics of two potentiometric sensors from the literature [3,24]. However, CPE generally have a number of advantages over PVC-based electrodes, as summarized in the work of various groups of authors [38,49,50]. In addition, the PVC-based sensor, which exhibited slightly improved response characteristics [24], contains the laboratory-intensive preparation of ionophore as electrode sensing material. On the other hand, the optimized CPE from this work, which is easy to prepare, is recommended for the determination of MAP because the expected concentrations of maprotiline in aliquots of urine samples and the drug tested should be within regular linear dynamic range.

## 4. Conclusions

This study describes an accurate and sensitive mCPE and its simple and relatively inexpensive preparation. The modified carbon paste electrode is prepared using MAP-TPB as the electro active ion-association complex as the electrode sensing material. The proposed CPE exhibits excellent selectivity, achieves Nernst slopes, has good reproducibility, and fast dynamic response time. It can be used in a wide maprotiline concentration range of 1.6 × 10^−7^–1.0 × 10^−2^ mol L^−1^ and in a wide pH range of 2.4–9.6. The recovery low relative standard deviations (especially by using the standard addition method) show high accuracy even at lower drug concentrations. The proposed CPE can be used as an alternative tool for the rapid quantification of maprotiline in pharmaceutical preparations and urine samples without significant matrix interference and with minimal sample pretreatment. It is therefore recommended for MAP determination in drug control laboratories.

## Figures and Tables

**Table 1:** CHN elemental analysis of ion-association complexes.

Ion-Association Complex		C (%)	H (%)	N (%)
MAP-TPB	Calculated	88.4	7.4	2.3
	Found	87.7	7.3	2.5
MAP_3_-PT	Calculated	19.4	2.0	1.1
	Found	19.8	2.2	1.0
MAP_3_-PM	Calculated	27.1	2.7	1.6
	Found	27.0	2.9	1.7
MAP-R	Calculated	48.3	5.1	16.4
	Found	48.5	5.0	16.4

**Table 2:** The response characteristics of prepared CPEs for potentiometric determination of maprotiline.

mCPE (no.)	Ingredient (%) ***						Linear Range (mol L^−1^)	Detection Limit **** (mol L^−1^)	Slope (mV/dec)± SD **	R^2^
	Binder		IAC		Salt					
*Efect of the type of binder*										
1	49.1 (DPB)		2.0	**MAP-TPB**	**0.1 (NaTPB)**		2.5 × 10^−6^–1.0 × 10^−2^	2.3 × 10^−6^	−50.3 ± 2.3	0.9907
2	49.0 (DOP)		2.1				2.5 × 10^−6^–1.0 × 10^−2^	2.2 × 10^−6^	−51.4 ± 1.9	0.9910
3	48.8 (DEHA)		2.0				1.6 × 10^−5^–1.0 × 10^−2^	1.6 × 10^−5^	−27.4 ± 3.9	0.9614
4	49.0 (PO)		1.9				-	-	−6.0 ± 3.9	0.6115
5	49.1 (TEPh)		2.0				6.3 × 10^−6^–4.0 × 10^−3^	5.8 × 10^−6^	−79.3 ± 3.8	0.9873
6 *	**49.0 (NPOE)**		**2.0**				**1.6 × 10^−7^–1.0 × 10^−2^**	**1.1 × 10^−7^**	**−59.5 ± 0.8**	**0.9979**
*Effect of differnet type and proportion of IACs*										
7	48.8	**NPOE**	0.7	**MAP-TPB**	**0.1 (NaTPB)**		1.0 × 10^−6^–1.0 × 10^−2^	9.7 × 10^−7^	−55.4 ± 1.1	0.9928
6 *	**49.0**		**2.0**				**1.6 × 10^−7^–1.0 × 10^−2^**	**1.1 × 10^−7^**	**−59.5 ± 0.8**	**0.9979**
8	48.8		5.3				2.5 × 10^−6^–1.6 × 10^−3^	1.9 × 10^−6^	−82.1 ± 2.8	0.9905
9	49.2		0.8	**MAP_3_-PT**			2.5 × 10^−6^–1.0 × 10^−2^	2.4 × 10^−6^	−52.3 ± 1.4	0.9913
10	48.9		2.1				1.0 × 10^−6^–4.0 × 10^−3^	9.2 × 10^−7^	−52.9 ± 1.4	0.9921
11	49.2		5.4				2.5 × 10^−6^–4.0 × 10^−3^	2.0 × 10^−6^	−52.1 ± 1.9	0.9948
12	49.0		0.8	**MAP_3_-PM**			6.3 × 10^−6^–4.0 × 10^−3^	6.0 × 10^−6^	−51.3 ± 2.4	0.9849
13	48.9		2.0				2.5 × 10^−6^–1.6 × 10^−3^	1.9 × 10^−6^	−51.9 ± 2.0	0.9900
14	49.1		5.2				1.6 × 10^−5^–4.0 × 10^−3^	1.6 × 10^−5^	−50.1 ± 0.9	0.9952
15	48.9		0.7	**MAP-R**			-	-	−13.8 ± 4.4	0.7808
16	49.2		2.2				-	-	−13.9 ± 6.9	0.6259
17	49.1		5.2				-	-	−12.8 ± 3.4	0.8041
*Effect of differnet type and proportion of ionic additive*										
6 *	**49.0**	**NPOE**	**2.0**	**MAP-TPB**	**0.1**	**NaTPB**	**1.6 × 10^−7^–1.0 × 10^−2^**	**1.1 × 10^−7^**	**−59.5 ± 0.8**	**0.9979**
18	49.0		2.0		0.3		2.5 × 10^−6^–4.0 × 10^−3^	2.3 × 10^−6^	−88.7 ± 3.2	0.9937
19	49.2		2.1		0.6		6.3 × 10^−6^–4.0 × 10^−3^	6.1 × 10^−6^	−89.1 ± 3.7	0.9871
20	48.8		2.1		0.1	TBATPB	2.5 × 10^−6^–1.0 × 10^−3^	2.2 × 10^−6^	−48.2 ± 1.6	0.9931
21	49.1		1.9		0.3		2.5 × 10^−6^–4.0 × 10^−3^	2.2 × 10^−6^	−48.0 ± 1.4	0.9919
22	49.1		2.0		0.7		6.3 × 10^−6^–1.0 × 10^−2^	5.7 × 10^−6^	−44.5 ± 1.7	0.9799

* Optimized carbon paste electrode (eleven replicates), ** standard deviation (five replicates), *** the proportion of graphite into the paste is the difference to the value of 100% of all paste ingredients; **** Detection limit is determined by extrapolating the calibration curve linear segments.

**Table 3:** Selectivity coefficients ($-logK_{THP,\;INT}^{pot})$ of various ions for proposed CPE.

Foreign Ions	$-logK_{MAP,\;INT}^{pot}$
K^+^	4.1
Na^+^	3.9
Li^+^	3.7
NH_4_^+^	4.2
Cu^2+^	3.6
Ca^2+^	3.8
Mg^2+^	3.8
Co^2+^	3.3
Zn^2+^	3.4
Al^3+^	3.5
Fe^3+^	3.0
L-arginine	4.2
L-cysteine	4.0
Lactose	3.7
Glucose	3.4
Galactose	3.6
Fructose	3.9
acetylsalicylic acid	3.8
Paracetamol	4.3
trihexyphenydil hydrochloride	2.9

**Table 4:** Analysis of MAP in pure solution, drug tablets, and spiked urine samples.

		Expected Data (mol L^−1^)	Recovery ± RSD * (%)
Pure solutions	Standard addition method	1.0 × 10^−4^	99.3 ± 0.5
		1.0 × 10^−5^	99.2 ± 0.6
		1.0 × 10^−6^	98.3 ± 2.1
	Potentiometric titration	1.0 × 10^−4^	99.0 ± 0.8
		1.0 × 10^−5^	99.0 ± 0.6
		1.0 × 10^−6^	95.2 ± 5.0
Ludiomil tablets	Standard addition method	1.0 × 10^−4^	100.5 ± 0.6
		1.0 × 10^−5^	100.7 ± 0.9
		1.0 × 10^−6^	98.1 ± 2.7
	Potentiometric titration	1.0 × 10^−4^	98.9 ± 0.8
		1.0 × 10^−5^	98.2 ± 1.4
		1.0 × 10^−6^	94.3 ± 5.4
Spiked urine samples	Standard addition method	1.0 × 10^−4^	98.3 ± 1.2
		1.0 × 10^−5^	97.9 ± 1.0
		1.0 × 10^−6^	103.1 ± 2.8
	Potentiometric titration	1.0 × 10^−4^	97.6 ± 1.4
		1.0 × 10^−5^	97.2 ± 1.6
		1.0 × 10^−6^	93.0 ± 4.2

* Five replicates.

**Table 5:** Statistical comparison between the potentiometric characteristics by the CPE prepared in this work and PVC-based sensors for maprotiline determination from the literature.

Parameter	Proposed CPE	PVC-Based Sensor [3]	PVC-Based Sensor with Ionophore [24]
slope (mV dec^−1^)	59.5 ± 0.8	55.4	59.1 ± 0.4
correlation coefficient	0.9979	0.9999	0.9934
limit of detection (mol L^−1^)	1.1 × 10^−7^	5.0 × 10^−6^	9.0 × 10^−10^
linear dynamic range (mol L^−1^)	1.6 × 10^−7^–1.0 × 10^−2^	1.0 × 10^−5^–1.0 × 10^−2^	1.0 × 10^−9^–1.0 × 10^−2^
response time (s)	<5	5	<5
working pH range	2.4–9.6	3.0–5.0	2.0–9.5
operating pH value	4.5 (acetate buffer)	3.5 (phosphate buffer)	3.1 (phosphate buffer)
average recovery in aliquot of Ludiomil tablets	100.7 ± 0.9 *	99.6 ± 4.4 ***	98.0 ± 2.0 ****
	98.1 ± 2.7 **		

* in aliquot of 1.0 × 10^−5^ mol L^−1^ of expected MAP concentration using the proposed standard addition method. ** in aliquot of 1.0 × 10^−6^ mol L^−1^ of expected MAP concentration using the proposed standard addition method. *** in aliquot of 8.1 × 10^−4^ mol L ^−1^ of expected MAP concentration^.^ **** the concentration of maprotiline in the aliquot in which the precision of the electrode was determined is not reported.
